# *Arabis watsonii* (P.H.Davis) F.K.Mey.: An overlooked cruciferous species from eastern Anatolia and its phylogenetic position

**DOI:** 10.3897/phytokeys.75.10568

**Published:** 2016-12-01

**Authors:** Barış Özüdoğru, Mehmet Fırat

**Affiliations:** 1 Department of Biology, Faculty of Science, Hacettepe University, Ankara Turkey; 2 Department of Biology, Faculty of Education, Yüzüncü Yıl University, Van, Turkey

**Keywords:** Arabis watsonii, Arabis hirsuta aggregate, Arabideae, Brassicaceae, phylogeny

## Abstract

*Arabis watsonii* (P.H.Davis) F.K.Mey. was initially reported as *Thlaspi watsonii* P.H.Davis in Flora of Turkey. Although F.K.Meyer transferred this species to *Arabis* L., this species has been overlooked and treated as *Thlaspi* L. in relevant literature for Flora of Turkey. In this study this species was evaluated using molecular (nuclear ITS and plastidic trnL-F sequences) and morphological data. Results clearly show that *Arabis watsonii* is sister to the *Arabis hirsuta* aggregate and its relatives. In conclusion, our results increased the number of known *Arabis* species in Turkey to 23. Furthermore, detailed description and distribution of the species are given and a new IUCN threat category for *Arabis watsonii* is proposed.

## Introduction

The genus *Arabis* L. is represented by ca. 60 species and distributed in temperate regions of the northern hemisphere (Koch et al. 2010, Al-Shehbaz 2012). Although Al-Shehbaz (1988) earlier delimited the genus with ca. 180 species, molecular phylogenetical studies of *Arabis* and its relatives clearly show that *Arabis* s.l. is polyphyletic (Koch et al. 1999, 2000, 2001, 2010, Al-Shehbaz et al. 2011) and subsequently some of the highly supported phylogenetic lineages were described as separate genera, e.g. *Scapiarabis* M.A. Koch, R. Karl, D. German & Al-Shehbaz, *Acirostrum* Y.Z.Zhao, and *Sinoarabis* R. Karl, D. German, M.A. Koch & Al-Shehbaz (Karl et al. 2012). Nevertheless, several highly supported *Arabis* clades (including main *Arabis* clade, *Arabis alpina* L. clade, *Arabis aucheri* Boiss. clade etc.) were also described (Karl and Koch 2013).

After the first revision of J. Cullen (1965) who reported 17 *Arabis* species in Flora of Turkey, five new species (*Arabis lycia* Parolly & P.Hein, *Arabis alanyensis* H.Duman, *Arabis davisii* H.Duman &A.Duran, *Arabis erikii* Mutlu and *Arabis kaynakiae* Daşkın) and two new records for Turkey (*Arabis allionii* DC. and *Arabis mollis* Steven) were added (Davis et al. 1988, Parolly and Hein 2000, Duman 2001, Duman and Duran 2001, Mutlu and Dönmez 2003, Mutlu 2004, Daşkın 2013). In addition, *Arabis graellsiiformis* Hedge was treated as a subspecies of *Arabis mollis* Steven (Mutlu and Erik 2012) and *Arabis turrita* L. transferred to *Pseudoturritis* Al-Shehbaz (Al-Shehbaz 2005). Finally, the genus *Arabis* is currently represented by 22 species (24 taxa) in Turkey (Mutlu and Erik 2015).

**Figure 1.**
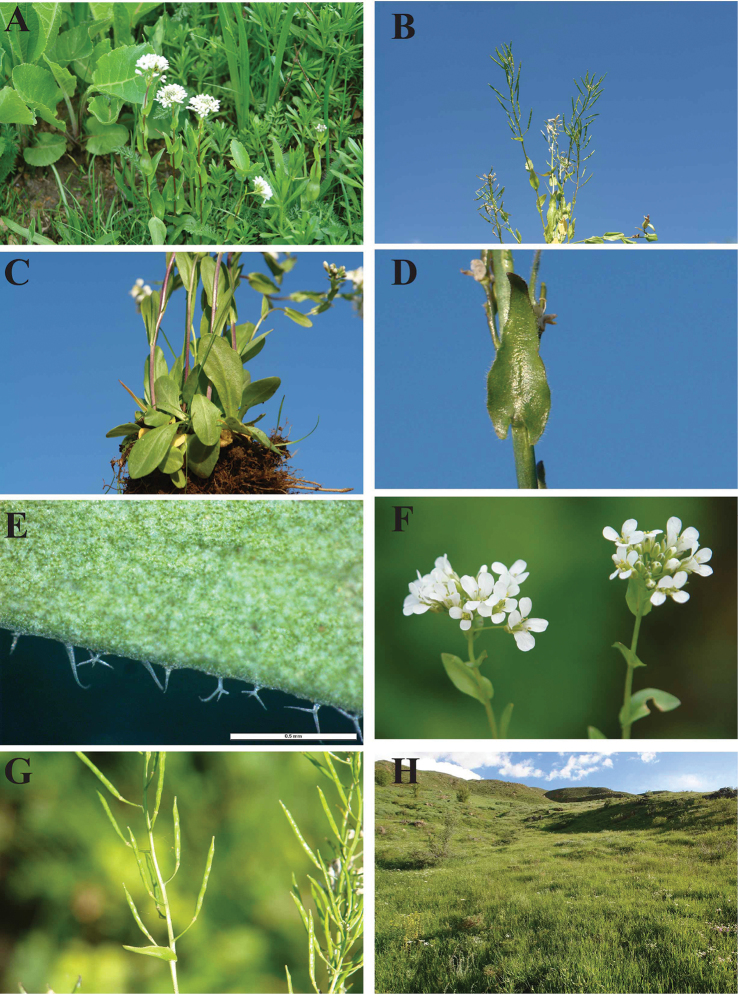
Photographs of *Arabis watsonii*, **A** habit (in flower) **B** habit (in fruit), **C** basal leaves **D** stem leaves **E** trichomes on leaves margins **F** flowers **G** fruits **E** habitat of Bahçesaray population.

*Arabis watsonii* (P.H.Davis) F.K.Mey. (Fig. 1) was initially published as *Thlaspi watsonii* P. H. Davis in Flora of Turkey by P. H. Davis. Davis argued that this taxon is closely related to the pink flowered *Thlaspi lilacinum* Boiss & Huet (*Callothlaspi lilacinum* (Boiss & Huet) F.K.Mey or *Noccaea lilacina* (Boiss. & A. Huet) Al-Shehbaz, depending on the authors and treatments). F.K. Meyer investigated type specimen of *Thlaspi watsoni* while working on his *Callothlaspi* account (Meyer 2006), and he transferred this species to *Arabis* since due to the presence of siliques (rather than silicles which are typical for *Thlaspi*) and leaves with bifid hairs. Ever since this taxon either has been overlooked (Al-Shehbaz et al. 2007, Özhatay et al. 2009, 2011, Mutlu and Erik 2015) or still treated as *Thlaspi* L. (Güner et al. 2014).

To verify Meyers’s treatment of *Arabis watsonii*, plant material was collected from type locality and nearby areas. Collected specimens were evaluated morphologically and molecularly to analyse 1) is *Arabis watsonii* a member of *Arabis* and 2), which main clade does it belong? We carried out morphological and molecular phylogenetic studies of *Arabis watsonii* plus representatives of *Arabis* and other Arabideae and combined this data with climatic and biogeographic data.

## Methods

### Sampling

The present study includes for the first time sequences of nuclear ribosomal ITS1, ITS2 and 5.8 S rRNA (hereafter ITS) and trnL(UAA) intron/trnL-trnF intergenic spacer sequence data (hereafter trnL-F) for *Arabis watsonii* (Voucher: M. Fırat 32513 at HUB). All other sequences of the 88 *Arabis* and other Arabideae species were taken from ITS and trnL-F data sets of Karl and Koch (2013). In addition, sequences of the related *Arabis hirsuta* aggregate (*Arabis stelleri* DC. and *Arabis takesimana* Nakai) were taken from GenBank (http://www.ncbi.nlm.nih.gov/genbank) and incorporated into the above mentioned data sets. To determine the phylogenetic placement of *Arabis watsonii*, we reduced the above mentioned data as follows: All *Arabis* species were added along with two species from *Aubrietia* Adans., two *Draba* L. species and one species each from other small genera including *Sinoarabis*, *Arcyosperma* O.E. Schulz, *Baimashania* Al-Shehbaz, *Acirostrum*, *Botschantzevia* Nabiev, *Dendroarabis* (C.A. Mey.) D. German & Al-Shehbaz, *Pachyneurum* Bunge, *Pseudodraba* Al-Shehbaz, D. German & M. Koch, *Scapiarabis*, *Tomostima* Raf. *Pseudoturritis turrita* (L.) Al-Shehbaz was used as the outgroup. Genbank accessions of species included in this study are at terminal nodes of phylogenetic trees (Figs 3, 4).

50 specimens belonging to five populations were used for extending description of *Arabis watsonii*. The vouchers were deposited at Hacettepe University Herbarium (HUB) and private herbarium of M. Fırat.

### DNA extraction, amplification and sequencing

Total genomic DNA was isolated using DNeasy Plant Mini Kit (Qiagen, Hilden, Germany) following the manufacturer’s instructions. ITS and trnL-F regions were amplified using primers ITS1 and ITS 4 (White et al. 1990) and C and F of Taberlet et al. (1991), respectively. Amplification of ITS and trnL-F followed the protocol in Warwick et al. (2004) and Ansell et al. (2007), respectively. Purification and sequencing were performed by BIOEKSEN (İstanbul, Turkey).

### Data analysis

Firstly, to determine whether *Arabis watsonii* belongs to tribe Arabideae, the phylogenetic tool in Brassibase (Koch et al. 2012; Kiefer et al. 2013) was used. ITS and trnL-F sequences data were edited with Codon Code Aligner (CodonCode Corporation) and directly incorporate into the alignment files of Karl and Koch (2013). Both data sets were analysed using a Bayesian approach as implemented in the software BEAST ver. 1.8 (Drummond et al. 2012).

Sequence evolution models were selected by the Akaike information criterion (AIC) implemented in MEGA v.6 (Tamura et al. 2013).

The GTR + G + I model was selected for ITS and GTR + I for trnL-F and a Yule process of speciation was used as the tree prior. Two independent Markov Chain Monte Carlo (MCMC) runs for each data set were conducted with 10 million generations and sampled every 1000 generations. Each run was checked using TRACER v1.6 (http://beast.bio.ed.ac.uk/Tracer) and then log and trees files were combined in LOGCOMBINER (Drummond et al. 2012).

A total of 20000 trees were obtained and 10% (2000) of these were discarded as burn-in. 18000 post-burn-in trees were used in the program TREEANNOTATER v.1.7.5 to obtain a single posterior probability and maximum clade credibility tree as visualized using FIGTREE v1.3.1.

### Morphology, distribution and conservation

To redescribe morphological features of *Arabis watsonii* ca. 50 individuals from five populations were investigated. In order to evaluate the IUCN thread category of *Arabis watsonii*, occurrence data were obtained from both field and Yüzüncü Yıl University, Science faculty herbarium (VANF) and then total distribution area was calculated using DIVA-GIS (version 7.5.0, http://www.diva-gis.org). Some specimens that had no GPS coordinates were georeferenced using Google Earth 7.1. (http://www.google.com/earth) according to common names. To evaluate climatic requirements of *Arabis watsonii* and closely related taxa, bioclimatic data were taken from the WorldClim–Global Climate Database (http://www.worldclim.org) at a spatial resolution of 30 s.

## Results

### Morphology, distribution and conservation

#### Arabis watsonii

Taxon classificationPlantaeBrassicalesBrassicaceae

(P.H.Davis) F.K.Mey (in Meyer 2006 p.187)

Arabis watsonii Basionym: Thlaspi watsonii P.H.Davis (in Davis et al. 1988 p. 235)

##### Type.

Turkey B9 Van: Çuh pass, Halanduran Da. and Güzeldere Tepe, dry stony slopes, locally common, 2800 m, flowers white, vi 1966, Albury, Cheese & Watson 1438 (holo. K photo !).

Slender perennial herb. Stem erect, nearly glabrose, 13–30 cm high. Basal leaves up to 30 mm, oblong – obovate, petiolate, leaves on sterile shoots completely covered by branched trichomes, fertile shoot with branched trichomes on the leaves margin. Stem leaves narrowly oblong, very shortly auriculate, tapering to subacute apex, 5–32 × 2.5–10 mm with marginal trichomes. Sepals white-purplish, inner sepals saccate, 3–3.2 × 1–1.8 mm, outer sepals smooth, 2.7–3 × 1–1.5 mm. Petals white, 5.5–7 mm long, 1.5 mm broad above, tapering below into 1.5–2 mm claw. Stamens 6, long filaments 4, 3–4 mm, short filaments 2, 2.5 – 3 mm long, anther yellow, 0.8 – 1 × 0.3–0.4 mm. Pedicel up to 7–8 mm in fruit. Fruit ± constricted between seeds, 4–14 × 0.8–1.2 mm, with 6–8 seeds, style ca. 1 mm. Seeds brown, ovate-oblong, 0.9–1.1 × 0.5–0.7 mm.

Fl. and fr : 4–6. Alpine damp places, dry stony slopes, steppe. 1980–2800 m.

##### Distribution:

**Figure 2.**
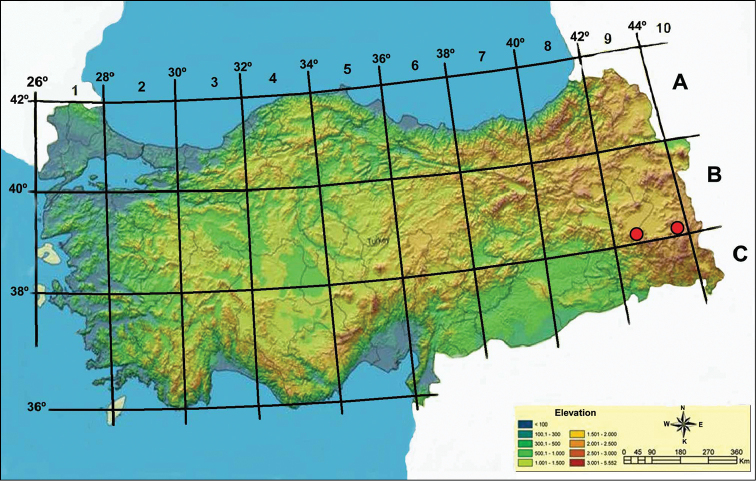
Distrubution of *Arabis watsonii* (red dots). Map was taken from Fırat (2016).

Endemic. Irano –Turanian element (Fig. 2)

##### Specimens examined.

**TURKEY. B9 Van**: Gürpınar district, from Güzeldere pass to Çuğ pass, damp places, 2495 m, 38°09'57"N, 43°57'47"E, 19 May 2015, M. Fırat 32513 (herb. M. Fırat); ibid M. Fırat 32572 (HUB, herb. M. Fırat) 11 June 2015; Bahçesaray district, from Ulubeyli village to Hizan, damp steppe, 2265 m, 38°07'46"N, 42°40'53"E, 19 May 2014, M. Fırat 30870 (HUB, herb. M. Fırat); ibid M. Fırat 30989 (HUB, herb. M. Fırat) 21 June 2014; Bahçesaray, between Liçan Village and Kavuşşahap Mountains (Deve Mountain), humid steppe, 1980 m, 04.04.1999. M. Fırat 1077 (herb. M. Fırat); Bahçesaray, Altındere Village Kavuşşahap Mountains, damp places, 2300 m, 17.05.2000, M. Fırat 2122 (herb. M. Fırat); Bahçesaray, between Cevizlibelen Village and Arnos Mountain, humid steppe,2400 m, 23.06.2000, *M. Fırat 2729* (herb. M. Fırat); between Güzelsu (Hoşap) and Başkale, Güzeldere pass, meadows, 2550–2650 m, 10.06.2001, M. Armağan *1073* (VANF); between Güzelsu (Hoşap) - Başkale, Güzeldere Pass, Güzeldere Hill, moist meadows, slopes, steppe, 2700–2800 m, 07.06.2002, M. Armağan 2423 (VANF); between Güzelsu (Hoşap) and Başkale, Güzeldere pass, from Güzeldere gendarmerie station to Başkale, slopes, steppe and moist meadows, 2600–2730 m, 19.05.2001, M. Armağan 1130 (VANF).

##### Vernacular name.

In Van province, indigenous people use name ‘Nançûk’ for *Arabis* species (Fırat 2013).

Field observations and records taken from relevant herbaria indicate that *Arabis watsonii* has two distinct populations (Fig. 2). A large population growing around Bahçesaray district and a second population occurring in the Gürpınar district especially around the Güzeldere pass. Therefore total distribution areas for these populations were estimated separately. The area around Bahçesaray was calculated as 108.99 km^2^ and the second area around Gürpınaras 2.69 km^2^. In summary, to propose IUCN threat categories of *Arabis watsonii*, these two population groups and calculated areas were considered. The occupancy area (AOO) of *Arabis watsonii* was calculated as 111.68 km^2^ in which about 1000 individuals in each population were estimated to occur. Overgrazing and reaping activities by the local people were observed in field studies. Therefore, in accordance with the criteria of the IUCN (2016), *Arabis watsonii* is assessed here as “Vulnerable” [(VU) (B2a, C2a(i))], because distribution area of the species is severely fragmented and the species is currently known from no more than ten localities occupying less than 2,000 km^2^, (although it was considered “Endangered” (EN) according to Ekim et al. (2000)).

The basic climatic requirements of *Arabis watsonii*, annual main temperature and annual precipitation were calculated as 5.7 °C and 583 ml respectively.

##### Phylogeny.

The aligned ITS and trnL-F data matrices included 91 species. The ITS data set was 642 bp, of which 236 were variable and 168 parsimony informative, whereas the trnL-F data set incorporates 855 bp, of which 181 were variable and 108 parsimony informative.

**Figure 3.**
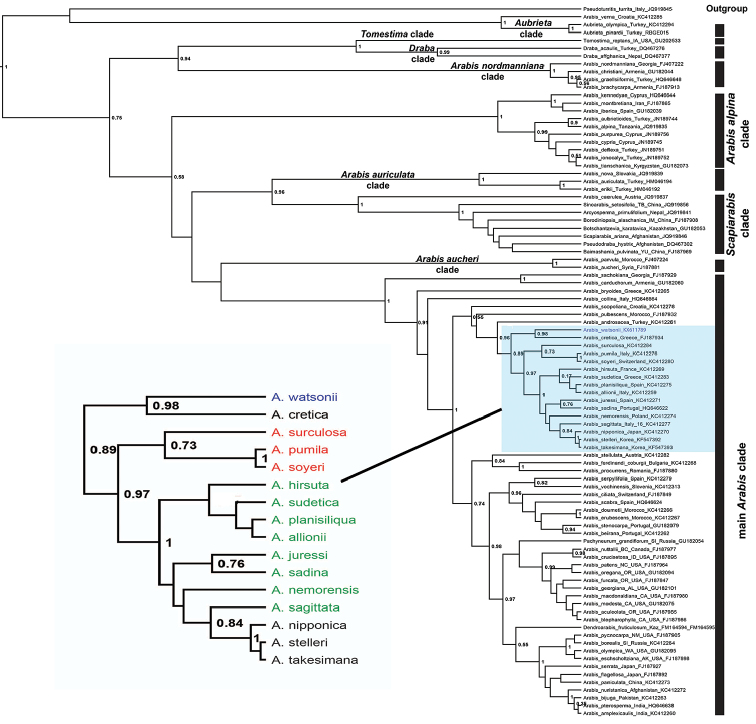
ITS-based phylogenetic backbone of Arabideae that focuses on the placement of *Arabis watsonii*. Shown is the Bayesian maximum clade credibility tree with posterior probability values > 0.5. Highlighted part of the tree, (magnified on the left), is the *Arabis hirsuta* aggregate and its relatives. Color codes: Green = *Arabis hirsuta* aggregate, red=the closest relatives of the *Arabis hirsuta* aggregate, blue = *Arabis watsonii*. Clade names follow Karl and Koch (2013).

**Figure 4.**
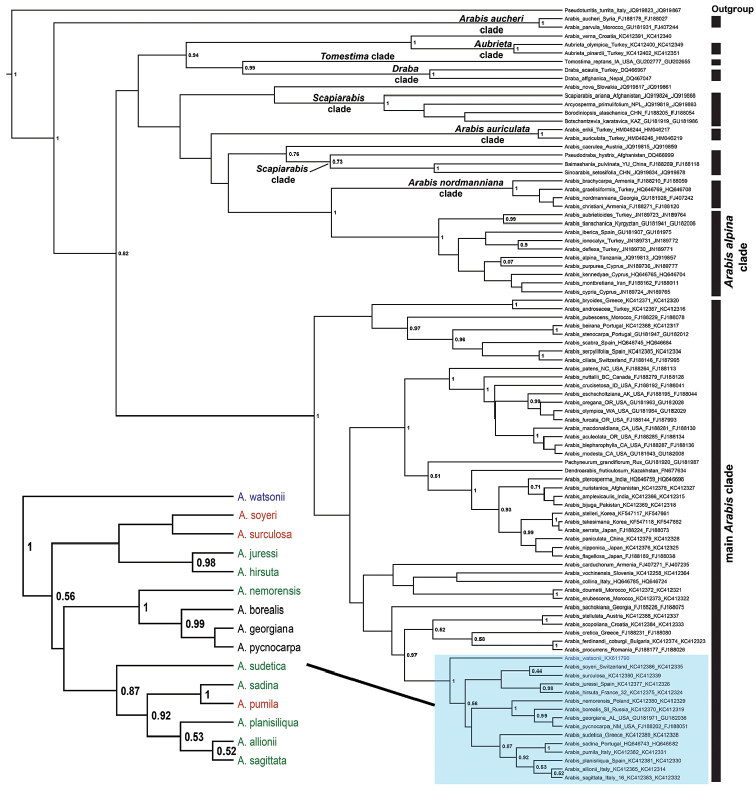
trnL-F-based phylogenetic backbone of Arabideae that focuses on the placement of *Arabis watsonii*. Shown is the Bayesian maximum clade credibility tree with posterior probability values > 0.5. Highlighted part of the tree, (magnified on the left), is the *Arabis hirsuta* aggregate and its relatives. Color codes: Green = *Arabis hirsuta* aggregate, red=the closest relatives of the *Arabis hirsuta* aggregate, blue = *Arabis watsonii*. Clade names follow Karl and Koch (2013).

The query of ITS sequences of *Arabis watsonii* in BrassiBase (version 1.1) supported its phylogenetic placement within tribe Arabideae and clearly matching *Arabis*. The outcome of Bayesian phylogenetic analyses using ITS and trnL-F data sets (Figs 3, 4) were congruent with each other in regard to the placement of *Arabis watsonii*. In both analyses *Arabis watsonii* falls into the main *Arabis* clade as sister to *Arabis hirsuta* (L.) Scop. aggregate and its relatives outlined in Karl and Koch (2014). Whereas *Arabis watsonii* forms a monophyletic lineage with *Arabis cretica* (Bayesian posterior probability (pp) = 0.98) in ITS analysis (Fig. 3), this sister relationship was not supported by trnL-F analysis (Fig. 4) and chloroplast data shows that *Arabis watsonii*, the *Arabis hirsuta* aggregate, its relatives plus non –European *Arabis* species are linked to this aggregate (including *Arabis georgiana* R.M.Harper, *Arabis pycnocarpa* M.Hopkins and *Arabis borealis* DC.) forming a monophyletic clade (pp=1.00).

## Discussion

In this study, we used evidence from nuclear ITS and plastidic trnL-F sequences to determine the phylogenetic and taxonomic position of the overlooked Anatolian endemic *Arabis watsonii*. In addition, morphological and climatic data were used to better understand ecological and evolutionary relationships of *Arabis watsonii* with representatives of the well-defined *Arabis hirsuta* aggregate and its relatives.

The differences in the phylogenetic placement of *Arabis watsonii* in relation to its sister position to *Arabis cretica*, according to ITS and trnL-F analyses, indicates possible chromosome capture /ancient hybridization. These processes are well known in the *Arabis hirsuta* aggregate and its relatives (Koch et al. 2010, Karl and Koch 2014).

A recent study of *Arabis hirsuta* aggregate recognised eight European species including *Arabis hirsuta* (L.) Scop., *Arabis sagittata* (Bertol.) DC., *Arabis planisiliqua* (Pers.) Rchb., *Arabis nemorensis* (Wolf ex Hoffm.) W.D.J. Koch, *Arabis allionii* DC., *Arabis sudetica* Tausch, *Arabis sadina* (Samp.) Coutinho, and *Arabis juressi* Rothm. The historical definition and circumscription of such an aggregate depends on different authors (see Karl and Koch (2014)) and because relationships between the *Arabis hirsuta* aggregate and its European/non-European relatives have already been discussed in detail before, we will not repeat this discussion here.

As indicated above, the ITS phylogeny supports a clear monophyly between *Arabis watsoni* and Greece endemic *Arabis cretica* Boiss. & Heldr. Whereas this relationship does not supported by trnL-F, both species seems to be at a basal position for *Arabis hirsuta* aggregate and its relatives. This results is somewhat expected because the Western Irano-Turanian and the East Mediterranean regions have already been suggested as diversity centres for almost all Arabideae clades (Jordon-Thaden et al. 2010, Karl and Koch 2013).

Morphologically *Arabis watsonii* is smilar to *Arabis hirsuta* and *Arabis sagittata*, therefore specimens were treated under these names in some herbaria. However *Arabis watsonii* differsfrom *Arabis hirsuta* and *Arabis sagittata* in having glabrous stems and relatively large petals. Meyer (2006) argued that *Arabis watsonii* is related to *Arabis abietina* Bornm. from the Ilgaz Mountain (Turkey) since this species is also characterized by glabrous stems and a similar petal length. The latter species has been treated as a synonym of *Arabis suedica* in Karl and Koch (2014) based on Jalas and Souminen (1994). Despite the relatively large geographic gap between *Arabis abietina* and *Arabis suedica*, ITS sequences of the taxa were identical and trnL-F sequences differ only in one single nucleotide position. Thus, *Arabis abietina* was not included in the current study, although it is treated as a valid species according to the Flora of Turkey (Cullen 1965) and the actual check-list (Güner et al. 2012). Apart from geographic isolation, branched trichomes on leaf margins and relatively small fruits are the main diagnostic characters distinguishing *Arabis watsonii* from both *Arabis abietina* and *Arabis allionii*, which are also members of the *Arabis hirsuta* aggregate. In summary, more comprehensive studies are needed to clarify the validity of *Arabis abietina*. With the proper assignment of *Arabis watsoni* in the current study, the total number of the Turkish *Arabis* species increased from 22 to 23 (25 taxa)

Finally, distribution in the alpine zone and perennial life cycle of *Arabis watsonii* is concordant with general trends of the tribe Arabideae (Karl and Koch 2013). Estimated climatic conditions for *Arabis watsonii*, including annual mean temperature, annual precipitation and other bioclimatic variable (not provided here) reflect a continental climate also described for other members of the *Arabis hirsuta* aggregate including *Arabis sudeica*, *Arabis hirsuta* etc. In conclusion, all environmental parameters and life cycle strategies of *Arabis watsonii* are in agreement with the genetic affiliation to the *Arabis hirsuta* aggregate and its relatives within the main *Arabis* clade.

## Supplementary Material

XML Treatment for Arabis watsonii
